# 692. Associations of nucleoside diphosphate-linked moiety X-type motif 15 mutations with neutropenia during antiviral therapy for cytomegalovirus disease or infection in pediatric liver transplant recipients

**DOI:** 10.1093/ofid/ofaf695.231

**Published:** 2026-01-11

**Authors:** Ken-ichi Iwata, Yuka Torii, Yuto Fukuda, Kazunori Haruta, Makoto Yamaguchi, Takako Suzuki, Yasuhiro Ogura, Jun-ichi Kawada

**Affiliations:** Department of Pediatrics, Nagoya University Graduate School of Medicine, Nagoya, Aichi, Japan; Nagoya University Graduate School of Medicine, Nagoya, Aichi, Japan; Department of Pediatrics, Nagoya University Graduate School of Medicine, Nagoya, Aichi, Japan; Nagoya University Graduate School of Medicine, Nagoya, Aichi, Japan; Nagoya University Graduate School of Medicine, Nagoya, Aichi, Japan; Nagoya University Graduate School of Medicine, Nagoya, Aichi, Japan; Department of Transplantation Surgery, Nagoya University Hospital, Nagoya, Aichi, Japan; Fujita Health University School of Medicine, Toyoake, Aichi, Japan

## Abstract

**Background:**

Cytomegalovirus (CMV) infection is a serious complication in pediatric liver transplant recipients, primarily due to their profoundly immunocompromised condition. Ganciclovir (GCV) and its oral prodrug valganciclovir (VGCV) are the standard antiviral agents employed for both prophylactic and therapeutic management of CMV-related conditions in this population. Nevertheless, neutropenia represents a common dose-limiting toxicity, which may necessitate temporary dose modification or discontinuation. Although genetic variants in the nucleoside diphosphate-linked moiety X-type motif 15 (NUDT15) gene are well-established predictors of thiopurine-induced hematologic toxicity, their involvement in GCV/VGCV-induced cytopenia remains underexplored.

**Figure:**
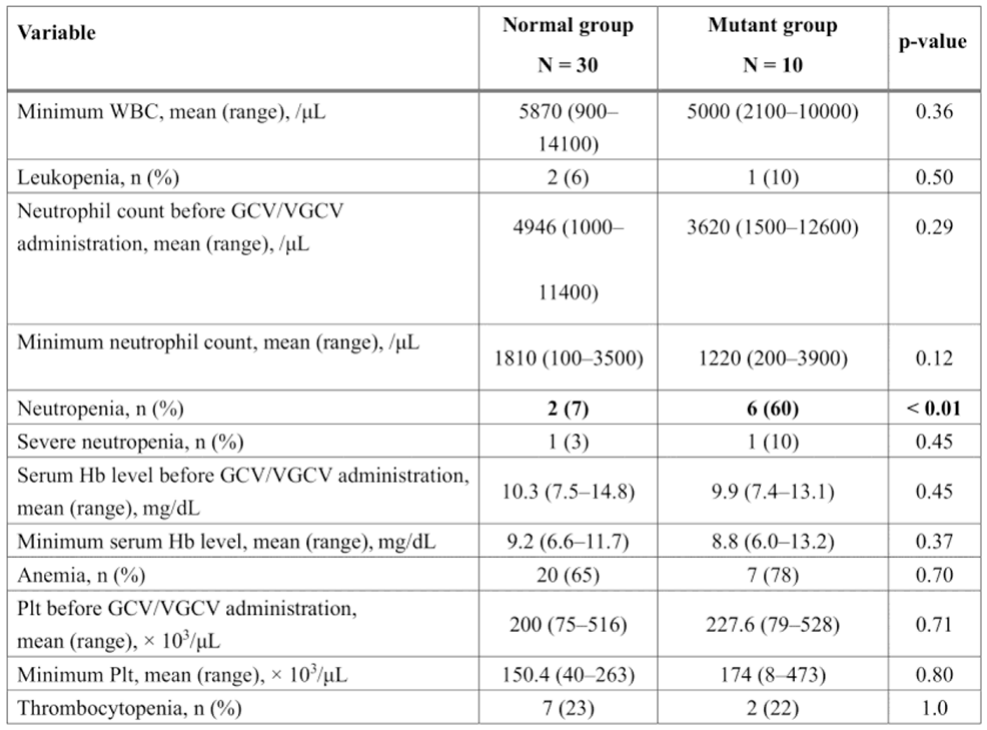
Decrease in blood cell counts and serum hemoglobin levels in each group

**Figure:**
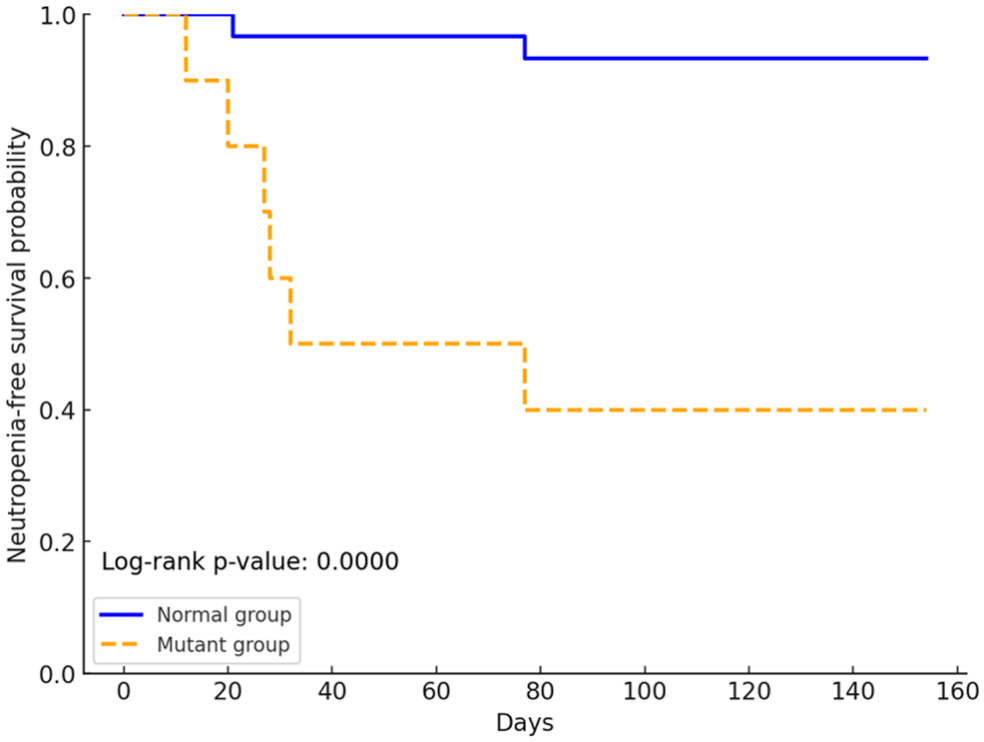
WBC, white blood cell count; GCV, ganciclovir; VGCV, valganciclovir; Hb, hemoglobin; Plt, platelet count Items with statistically significant differences (p < 0.05) are shown in bold font. Kaplan–Meier curves representing neutropenia-free survival during antiviral therapy The time from the initiation of antiviral therapy to the onset of neutropenia is shown using Kaplan–Meier curves for the normal and mutant groups. Neutropenia, neutrophil count < 1000/μL

**Methods:**

In this single-center retrospective cohort study, we analyzed clinical records of pediatric liver transplant recipients who received GCV or VGCV for confirmed or suspected CMV infection at Nagoya University Hospital between 2012 and 2022. All patients underwent genotyping for NUDT15 variants (R139C, R139H, V18I). Hematologic data and adverse events were compared between wild-type and variant groups.

**Results:**

Of the 40 patients included, 10 (25%) carried NUDT15 variants. Neutropenia was observed in 6 /10 cases (60%) of mutation group and 2 /30 cases (7%) of normal group (p< 0.01). The median neutrophil count reduction was greater in the mutation group (−65.1% vs. −45.9%, p=0.02). Neutropenia-free survival was significantly prolonged in the normal group (log-rank test, p < 0.01). The mean number of days from the initiation of GCV/VGCV treatment to the onset of neutropenia was 49 days (21–77 days) for the normal group and 32.7 days (12–77 days) for the mutant group (median, 27.7 days).

**Conclusion:**

These findings support the integration of pharmacogenetics into clinical practice. Early identification of high-risk individuals through genetic screening may guide antiviral selection, dosing, and monitoring. Tailoring therapy to host genomics may reduce complications and improve outcomes. Incorporating NUDT15 genotyping into routine transplant protocols offers a practical application of precision medicine in pediatric care.

**Disclosures:**

All Authors: No reported disclosures

